# Construction and evaluation of STZ-induced diabetes and diabetic kidney disease models in C57BL/6J mice

**DOI:** 10.3389/fendo.2025.1711035

**Published:** 2025-12-17

**Authors:** Hao Li, Fang Qin, Shanshan Zheng, Jing Wu, Sen Lin, Xinyuan Gao, Yipeng Liu

**Affiliations:** Department of Nephrology, The First Affiliated Hospital of Shandong First Medical University and Shandong Provincial Qianfoshan Hospital, Shandong Institute of Nephrology, Jinan, Shandong, China

**Keywords:** C57BL/6J mice, diabetes mellitus, diabetic nephropathies, models animal, streptozotocin

## Abstract

**Objective:**

To explore the optimal strategy for streptozotocin (STZ)-induced models of diabetes mellitus (DM) and Diabetic kidney disease (DKD) in C57BL/6J mice, and to analyze potential factors influencing model stability.

**Methods:**

Forty-five 6-week-old male C57BL/6J mice were randomly assigned to six groups: normal control (CON), unilateral nephrectomy control (CU), standard diet with STZ (CS), standard diet with unilateral nephrectomy and STZ (CUS), high-fat diet with STZ (HS), and high-fat diet with unilateral nephrectomy and STZ (HUS). Mice in the HS and HUS groups received a high-fat diet (HFD) for 6 weeks, followed by unilateral nephrectomy (UN) or sham surgery, and were then administered STZ (35 or 45 mg/kg/day, intraperitoneally) for five consecutive days to induce DM. DM induction was confirmed when two consecutive random blood glucose (RBG) measurements were ≥ereu mmol/L. Throughout the study, RBG, body weight, and urine albumin-to-creatinine ratio (UACR) were monitored longitudinally. At 19 weeks post-induction, mice were euthanized for kidney weight assessment and histopathological examination.

**Result:**

All STZ-treated mice initially developed diabetes (100%); however, sustained hyperglycemia was not achieved in all cases. Glycemic stability was strongly influenced by the induction strategy (P<0.05). Specifically, 45 mg/kg/day STZ with a normal diet yielded only a 14.3% remission rate (2/14), whereas 35 mg/kg/day STZ with a HFD resulted in a 62.5% remission rate (10/16). Although 45 mg/kg/day STZ combined with a HFD maintained persistent hyperglycemia, it was accompanied by excessive mortality (80%, 8/10). UN was not associated with glycemic stability (P > 0.05); however, it markedly accelerated DKD progression and exhibited a synergistic effect with HFD. Furthermore, compared with mice exhibiting partial glycemic remission, those with stable hyperglycemia demonstrated significantly higher kidney weight, kidney-to-body weight ratio, and UACR (P<0.05).

**Conclusion:**

An appropriate dose of STZ in combination with UN and HFD represents an optimal strategy for establishing an STZ-induced DKD model in C57BL/6J mice, effectively recapitulating the clinical and pathological features of human DKD and providing a robust platform for mechanistic research and therapeutic development.

## 1 Introduction

The global prevalence of diabetes mellitus (DM) is increasing at an unprecedented rate. The International Diabetes Federation (IDF) estimates that 589 million adult individuals were affected in 2024, a number projected to rise to 853 million by 2050 (1). Diabetic kidney disease (DKD), the most common and destructive microvascular complication of DM, develops in 30–40% of patients and has become the leading cause of end-stage renal disease (ESRD) worldwide (2–4). Despite extensive research, the mechanisms underlying DKD remain incompletely defined, underscoring the need for stable and reproducible animal models to advance mechanistic studies and therapeutic discovery.

DKD models are broadly classified into induced, spontaneous, and genetically engineered types. While spontaneous and transgenic models recapitulate human phenotypes more closely, their high cost and technical complexity limit wide application. In contrast, induced models are easier to establish and remain the mainstay of experimental research. Streptozotocin (STZ), a widely used diabetogenic compound, selectively enters pancreatic β-cells via glucose transporter 2 (GLUT2) and induces apoptosis through DNA alkylation (5). Owing to its chemical stability and low direct toxicity, STZ has become the cornerstone for generating diabetes and DKD models.

C57BL/6J (B6) mice are commonly employed in modeling human diseases, including DKD, owing to their affordability, robust breeding, and amenability to genetic manipulation (6). Although they are relatively resistant to DKD compared with strains such as DBA/2J or KK/HIJ (7, 8), B6 mice remain the preferred background for mechanistic research. Prior studies suggest that combining unilateral nephrectomy (UN), high-fat diet (HFD), and STZ injection accelerates diabetes induction in B6 mice (9), yet the extent of renal injury under these conditions is not fully defined. Other reports indicate that UN combined with STZ induces type 1 DKD under standard diet but fails to generate type 2 DKD under HFD (10).

In our study, we observed that UN accelerates DKD progression without significantly affecting baseline body weight, blood glucose, or RBG ≥16.7 mmol/L (UACR). UN induced only compensatory hypertrophy of the contralateral kidney, with no overt renal injury or impact on model stability. Glycemic remission observed in some diabetic mice was likely a consequence of inadequate STZ dosing. Notably, the combination of UN and STZ successfully established DKD in B6 mice under both standard and HFD conditions, with more severe renal pathology evident in the HFD group.

Based on these findings, we systematically compared the effects of UN, HFD, and STZ in different combinations on the development of DKD in B6 mice, evaluated model stability and pathological features, and analyzed potential factors affecting reproducibility. These findings provide a robust and reproducible experimental platform for dissecting DKD pathogenesis and for preclinical therapeutic testing.

## 2 Materials and methods

### 2.1 Experimental animals and workflow

Forty-five male SPF B6 mice (6 weeks old, 20.70 ± 0.90g) were obtained from Jicui Yaokang Biotechnology Co., Ltd. (Jiangsu, China; SCXK(Su)202-0009). Mice were housed 5 per ventilated cage under standard conditions (12 h light/dark cycle, 22 ± 2°C, 50 ± 10% relative humidity) with ad libitum access to food and water.

Following a 2-week acclimation, mice were randomly assigned to six groups (n=5–15): normal control (CON, n=5), unilateral nephrectomy control (CU, n=5), standard diet + STZ (CS, n=5), standard diet + unilateral nephrectomy + STZ (CUS, n=5), high-fat diet + STZ (HS, n=10), and high-fat diet + unilateral nephrectomy + STZ (HUS, n=15). CON, CU, CS, and CUS groups received standard maintenance chow (No. 1002, Xiahe Technology Development, Pizhou, China), while HS and HUS received a 60 kcal% high-fat purified diet (No. 10060, Sino Biological Technology, Siping, China). After six weeks, mice underwent UN or sham surgery. One week later, diabetes was induced by intraperitoneal injection of STZ (S8050, Solarbio, China) or citrate buffer (C1013, Solarbio, China). In preliminary studies, 45 mg/kg/day STZ caused 80% mortality in HUS mice, despite marked DKD pathology in surviving mice (**Supplementary Table 1**), so the dose was reduced for HFD groups.

Mice were fasted for 12 h (water ad libitum) before STZ injection, administered for five consecutive days. Seventy-two hours after the last injection, random blood glucose (RBG) was measured via tail vein sampling. Mice with two consecutive readings ≥16.7 mmol/L were considered diabetic (11–13). The day of successful induction was designated week 0 (0 w) for experimental tracking.

**Figure 1:**
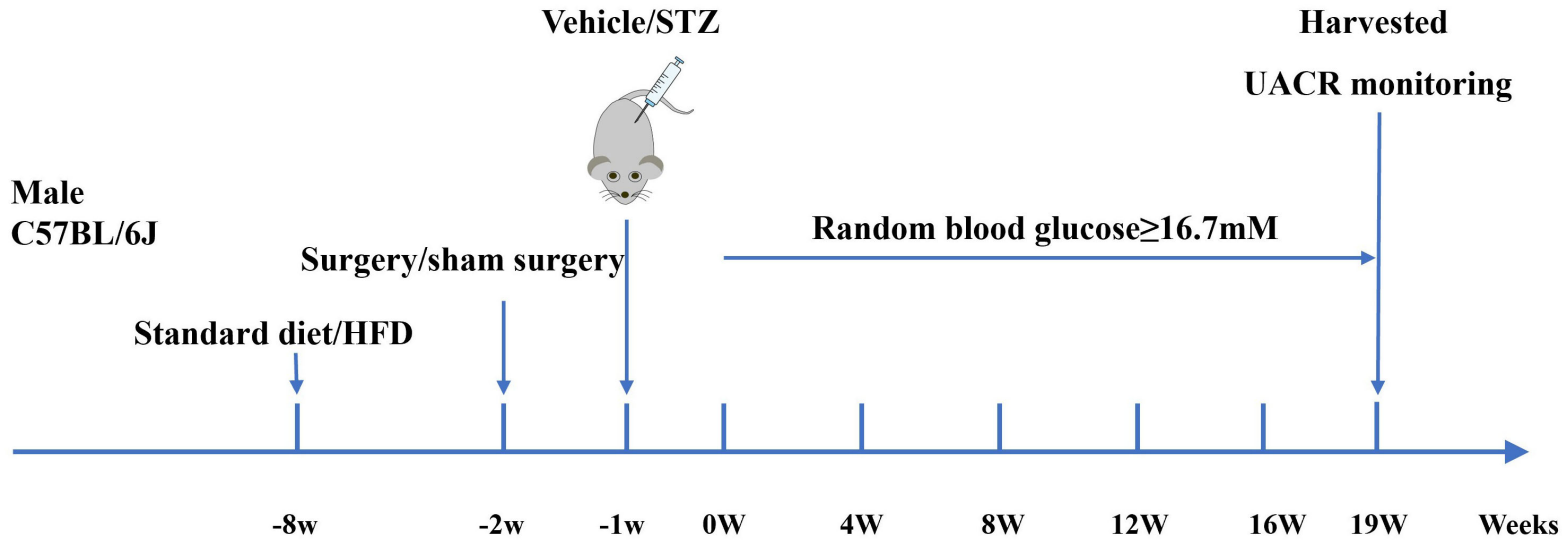
Workflow for establishing the diabetic mouse model. The day of successful diabetes induction was designated as week 0 (0W). HFD, high-fat diet; STZ, streptozotocin; UACR, urine albumin-to-creatinine ratio.

All procedures were approved by the Animal Ethics Committee of the First Affiliated Hospital of Shandong First Medical University (2023111501). Details of mouse grouping and model establishment are provided in **Table 1** and **Figure 1**.

**Table 1:** Experimental design and interventions in mice.

Groups	Feeding	Treatment	Drug and dosage
CON(n=5)	Standard diet	Sham	Citrate buffer (0.1M)
CU(n=5)	Standard diet	UN	Citrate buffer (0.1M)
CS(n=5)	Standard diet	Sham	STZ (45mg/kg/d *5d)
CUS(n=5)	Standard diet	UN	STZ (45mg/kg/d *5d)
HS(n=10)	HFD	Sham	STZ (35mg/kg/d *5d)
HUS(n=15)	HFD	UN	STZ (35mg/kg/d *5d)

CON, normal control; CU, unilateral nephrectomy control; CS, STZ-induced diabetic mice on standard diet; CUS, STZ + unilateral nephrectomy on standard diet; HS, STZ-induced diabetic mice on high-fat diet; HUS, STZ + unilateral nephrectomy on high-fat diet. STZ, streptozotocin; UN, unilateral nephrectomy; HFD, high-fat diet.

### 2.2 Unilateral nephrectomy

**Figure 2:**
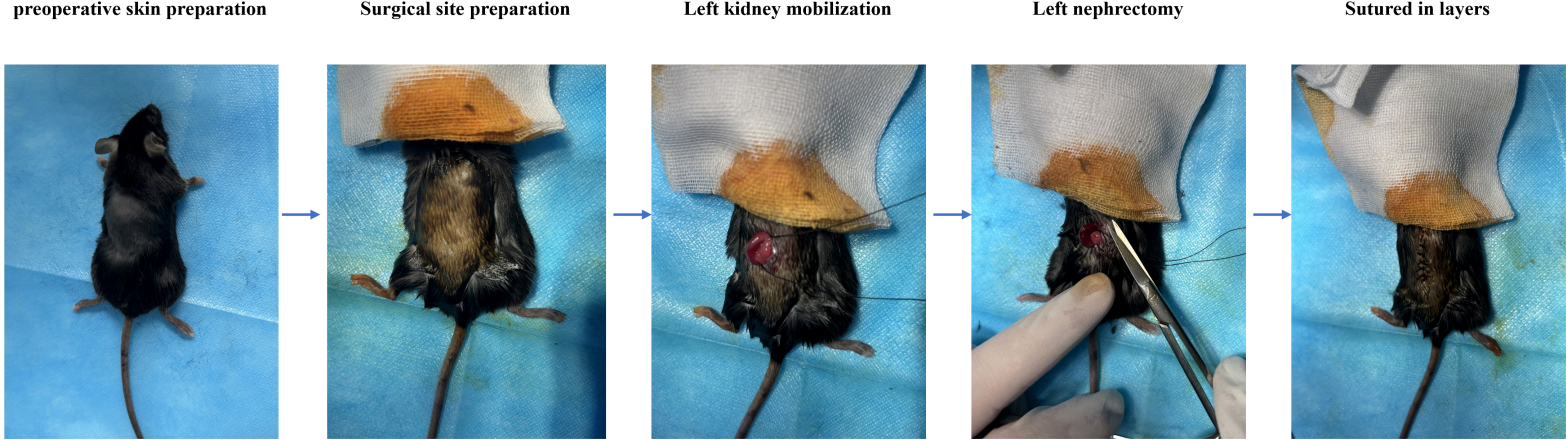
Left nephrectomy procedure in mice.

Before the operation, skin was prepared for the mice. UN was performed under isoflurane inhalation anesthesia. Mice were initially anesthetized in an induction chamber with 2.0% isoflurane in oxygen at an oxygen flow rate of 0.5-1.0 L/min until loss of the righting reflex. After induction, mice were transferred to a surgical platform and maintained under 1.5% isoflurane in oxygen via a nose cone throughout the procedure. Depth of anesthesia was monitored by assessing respiratory rate, heart rate, and loss of pedal reflex to toe pinch. Following anesthesia, the mice were placed in the prone position and the surgical site was disinfected with povidone-iodine. A transverse incision of approximately 1 cm was made 0.5 cm lateral to the spine at the intersection of the ear–tail line and the lower margin of the left costal arch. The muscle layers were bluntly dissected to expose the left kidney. Renal vessels were individually ligated with 4–0 absorbable sutures, and the kidney was excised. The muscle and skin were then closed in layers using 3–0 absorbable sutures. After surgery, mice were placed on a warming pad until recovery of spontaneous activity. For three days postoperatively, the incision site was disinfected with povidone-iodine twice daily. One mouse died intraoperatively and one postoperatively, yielding a surgical success rate of 92%. The procedure is illustrated in **Figure 2**.

### 2.3 STZ injection

STZ solution was freshly prepared immediately prior to use. Mice were fasted overnight and weighed, and the required amount of STZ (lyophilized powder) was calculated based on the number of animals and intended dose. The STZ powder was placed in a sterile 1.5 mL microcentrifuge tube and wrapped in aluminum foil to protect from light. Both the tube containing STZ and 0.1 M citrate buffer (pH 4.5) were pre-chilled in an ice bath and brought to the animal facility. STZ was dissolved in citrate buffer at 1% (w/v), mixed thoroughly, and sterilized through a 0.22 µm filter (SLGP033RB, Merck) to obtain the injection solution. Each mouse received the calculated volume based on its fasting body weight, and all injections were completed within 30 minutes.

### 2.4 Blood glucose, body and kidney weight measurements, and urine collection

Body weight and RBG was monitored regularly throughout the study. RBG levels were measured via tail vein sampling using a glucometer (Youankang, JZ Medical Technology Co., Ltd., China). At week 19 post-modeling, mice were housed individually in metabolic cages (Tecniplast, Italy) for 24-hour urine collection. After urine collection, mice were anesthetized and subjected to thoracotomy and laparotomy (as described above). The right atrium was incised and the animals were perfused via the left ventricle with physiological saline until effluent ran clear. Kidneys were excised, weighed, and a portion was fixed for transmission electron microscopy; remaining tissue was fixed in 4% paraformaldehyde.

For euthanasia, mice were euthanized under deep inhalational anesthesia with 5.0% isoflurane in oxygen until respiratory and cardiac arrest occurred, and exposure in the chamber was continued for an additional 60 s to ensure cessation of respiration. Death was confirmed by absence of heartbeat, respiration, and reflex responses (e.g., no response to a toe-pinch).

### 2.5 Measurement of urinary creatinine and albumin by ELISA

Twenty-four-hour urine samples were collected and centrifuged at 1,000 × g for 20 min at 4 °C to remove cellular debris. The resulting supernatants were collected for analysis. Urinary albumin was measured using a mouse urine microalbumin ELISA kit (E-EL-M0792, Elabscience, China) after diluting the supernatants 1,000–3,000-fold. Urinary creatinine was determined directly from undiluted supernatants using a creatinine ELISA kit (RE10024, ReedBiotech, China).

### 2.6 Histological analysis

Kidney tissues fixed in 4% paraformaldehyde for 48 h were dehydrated through a graded ethanol series and embedded in paraffin. Paraffin blocks were sectioned at 3 μm, deparaffinized, and rehydrated. Sections were stained with hematoxylin and eosin (HE, Cat: G1121, Solarbio) or periodic acid–Schiff (PAS, Cat: G1280, Solarbio) and examined under a light microscope.

### 2.7 Statistical analysis

Data were analyzed using SPSS 25.0. Normality of distribution was assessed by the Shapiro–Wilk test. For normally distributed data, one-way ANOVA was applied; Bonferroni correction was used for *post hoc* multiple comparisons when variances were equal, whereas Dunnett’s T3 test was applied if variances were unequal. Non-normally distributed data were analyzed using the Kruskal–Wallis H test with Bonferroni-adjusted *post hoc* comparisons. All figures were generated with GraphPad Prism 10.1.2, and P < 0.05 was considered statistically significant.

## 3 Results

### 3.1 Rate of successful model induction, glycemic relapse, and mortality in mice

Forty-five male B6 mice were initially enrolled, of which five died during the pre- and post-operative STZ treatment, leaving 40 mice for analysis. After excluding the normal control and UN control (n = 9), 31 mice received STZ injections. All treated mice initially met the diabetes induction criteria (two consecutive RBG ≥16.7 mmol/L, 100% initial success). During the follow−up period, sustained hyperglycemia was observed in 15 mice, whereas 13 animals showed spontaneous improvement in blood glucose levels. Among the latter, three mice died of undetermined causes (one in the HS group and two in the HUS group). Overall, persistent hyperglycemia was maintained in 48.4% of the cohort, the spontaneous remission rate was 41.9%, and the mortality rate was 9.7% (CS: 0%; CUS: 0%; HS: 11.1%; HUS: 13.3%). Notably, previous studies have shown that mice with such spontaneous glycemic recovery still experience β-cell loss and impaired pancreatic islet function and are therefore considered diabetic (14, 15). While the overall induction of the diabetes model was considered fully successful, the confirmed persistence rate of hyperglycemia was 48.4%. This divergence primarily stems from issues related to subsequent blood sample quality, which precluded reliable measurement of serum insulin and C-peptide levels. As a result, it remains undetermined whether mice that showed spontaneous glycemic remission continued to exhibit insulin resistance or impaired insulin secretion. Although these animals maintained classic diabetic phenotypes—including polydipsia, polyphagia, and polyuria—the lack of biochemical data on insulin and C-peptide limits a comprehensive assessment of their ongoing diabetic status and underlying metabolic abnormalities. According to the recommendations of the Animal Models of Diabetic Complications Consortium (AMDCC), mice can only be considered to exhibit features comparable to early human DKD if they present with persistent hyperglycemia, marked proteinuria, and corresponding renal pathological changes (16). Consequently, mice with spontaneously improved blood glucose in this study were not classified as DKD. Ultimately, 15 mice developed DKD, yielding a final modeling success rate of 48.4% (**Supplementary Table 2**).

### 3.2 General condition of mice

CON and CU mice appeared healthy, with smooth, shiny fur, normal activity, stable body weight, and regular food and water intake. In contrast, STZ-injected mice developed classic diabetic symptoms—polydipsia, polyphagia, and polyuria—approximately two weeks after model induction. At later stages, these mice showed mild lethargy, occasional hair loss, wet fur, and increased time spent lying down.

### 3.3 Random blood glucose, body weight, kidney weight, UACR, histopathology, and model evaluation

#### 3.3.1 Random blood glucose dynamics across groups

**Figure 3:**
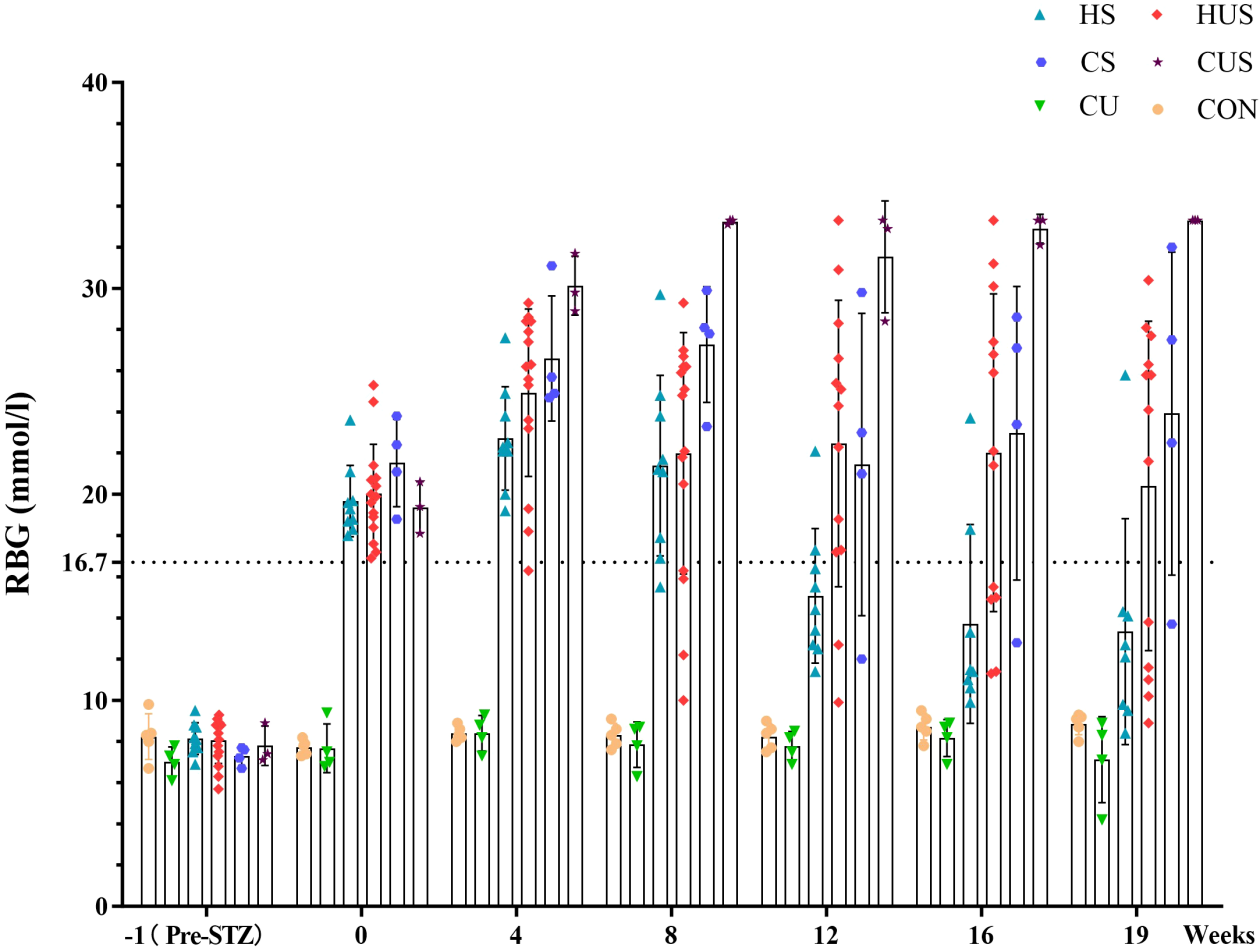
RBG dynamics in each experimental group. CON, normal control; CU, unilateral nephrectomy control; CS, STZ-induced diabetic mice on standard diet; CUS, STZ + unilateral nephrectomy on standard diet; HS, STZ-induced diabetic mice on high-fat diet; HUS, STZ + unilateral nephrectomy on high-fat diet. Throughout the modeling period, mice with RBG ≥ 16.7 mmol/L were considered diabetic.

At baseline, RBG levels were comparable across groups, and UN did not influence glycemic status. Following five consecutive days of intraperitoneal STZ administration, all 31 treated mice met the induction criteria for diabetes (RBG ≥16.7 mmol/L). During follow-up, however, 16 mice showed spontaneous declines in RBG below this threshold, predominantly in the HS group, and 3 of these mice died of unknown causes. Ultimately, 15 mice maintained stable hyperglycemic levels. Longitudinal changes in RBG across groups are shown in **Figure 3**.

#### 3.3.2 Body weight changes in each group of mice

**Figure 4:**
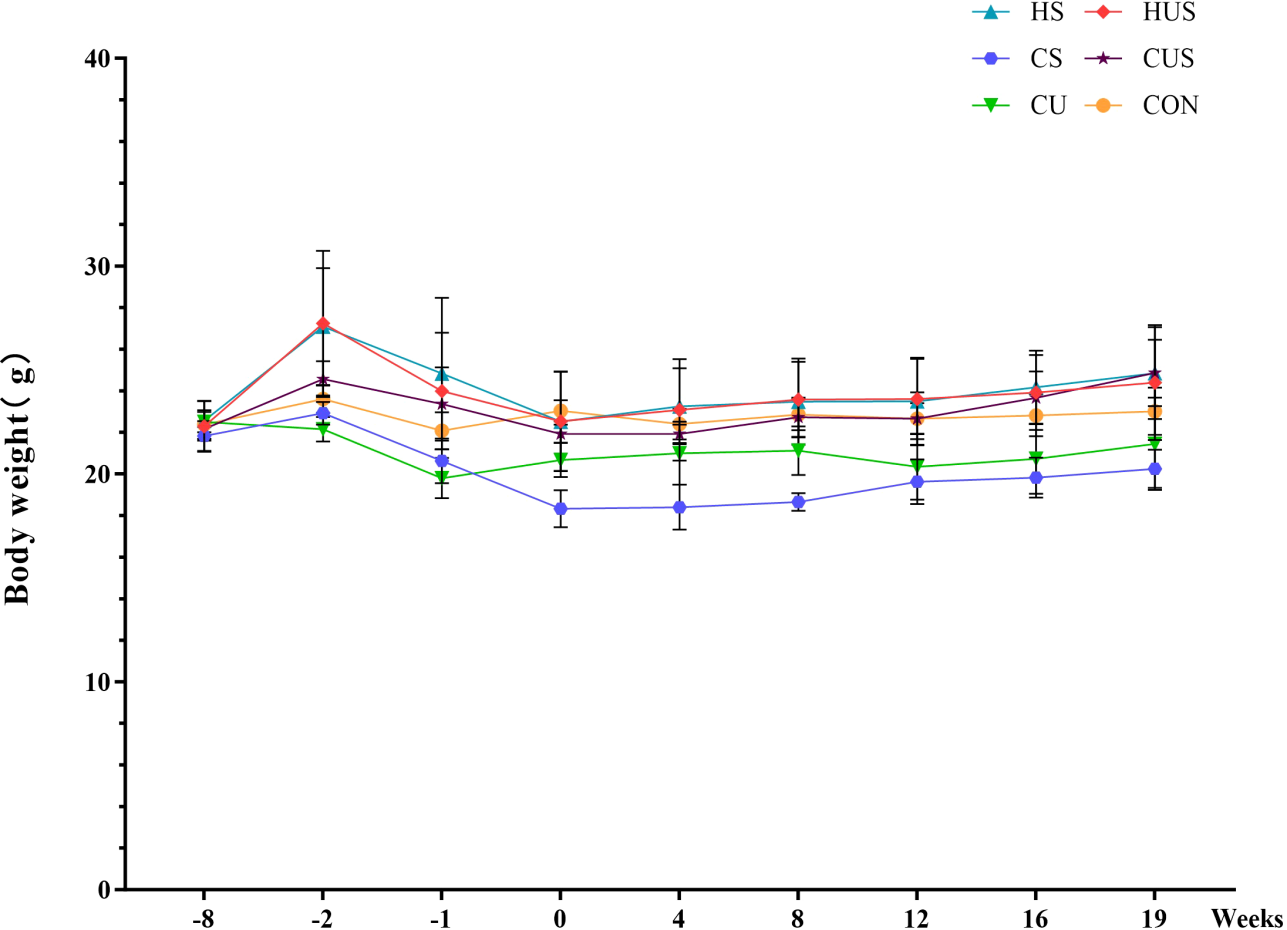
Temporal changes in body weight across experimental groups.

Before the introduction of different diets, there were no significant differences in body weight among the groups (P > 0.05). After 6 weeks of HFD (60 kcal% fat), mice in the HUS and HS groups exhibited a significant increase in body weight (P < 0.05). However, following UN or sham surgery and intraperitoneal injection of STZ or citrate buffer, all groups experienced a marked transient body weight loss, after which body weight remained relatively stable. Changes in body weight for each group are shown in **Figure 4** and **Table 2**.

**Table 2:** Temporal changes in body weight across experimental groups.

Weeks Groups	-8w	-2w	-1w	0w	4w	8w	12w	16w	19w
CON	22.38 ± 0.69	23.60 ± 0.65	22.08 ± 0.89	23.04 ± 0.51	22.40 ± 0.73	22.86 ± 0.74	22.66 ± 0.74	22.82 ± 1.01	23.02 ± 1.13
CU	22.50 ± 0.48	22.15 ± 0.59	19.80 ± 0.96	20.68 ± 0.83^#^	21.00 ± 1.52	21.13 ± 1.17	20.35 ± 1.59	20.73 ± 1.68	21.45 ± 2.22
CS	21.83 ± 0.72	22.93 ± 0.54	20.63 ± 1.07	18.33 ± 0.89^##^	18.40 ± 1.08^#^	18.65 ± 0.42^##^	19.63 ± 1.07	19.83 ± 0.96	20.25 ± 0.92
CUS	22.17 ± 0.21	24.57 ± 0.86	23.37 ± 1.76	21.93 ± 0.45	21.93 ± 0.45	22.73 ± 0.95	22.67 ± 1.27	23.67 ± 1.27	24.87 ± 1.59
HS	22.68 ± 0.94	27.69 ± 2.31^#^	25.36 ± 3.51	22.79 ± 2.41	23.55 ± 1.71	23.85 ± 1.86	23.84 ± 1.96	24.18 ± 1.75	24.85 ± 2.21
HUS	22.46 ± 1.22	27.95 ± 3.16^##^	24.58 ± 2.51	22.73 ± 2.52	23.32 ± 2.51	23.84 ± 1.79	23.61 ± 1.93	23.92 ± 1.81	24.39 ± 2.77
F	0.475	10.537	4.905	3.964	5.352	8.950	5.705	6.626	3.653
P	0.792	0.000	0.002	0.007	0.001	0.000	0.001	0.000	0.010

Data are expressed as mean ± SD. Compared with the CON group: ^#^P < 0.05, ^##^P < 0.01.

#### 3.3.3 Comparison of kidney weight, kidney-to-body weight ratio, and UACR among experimental groups

**Figure 5:**
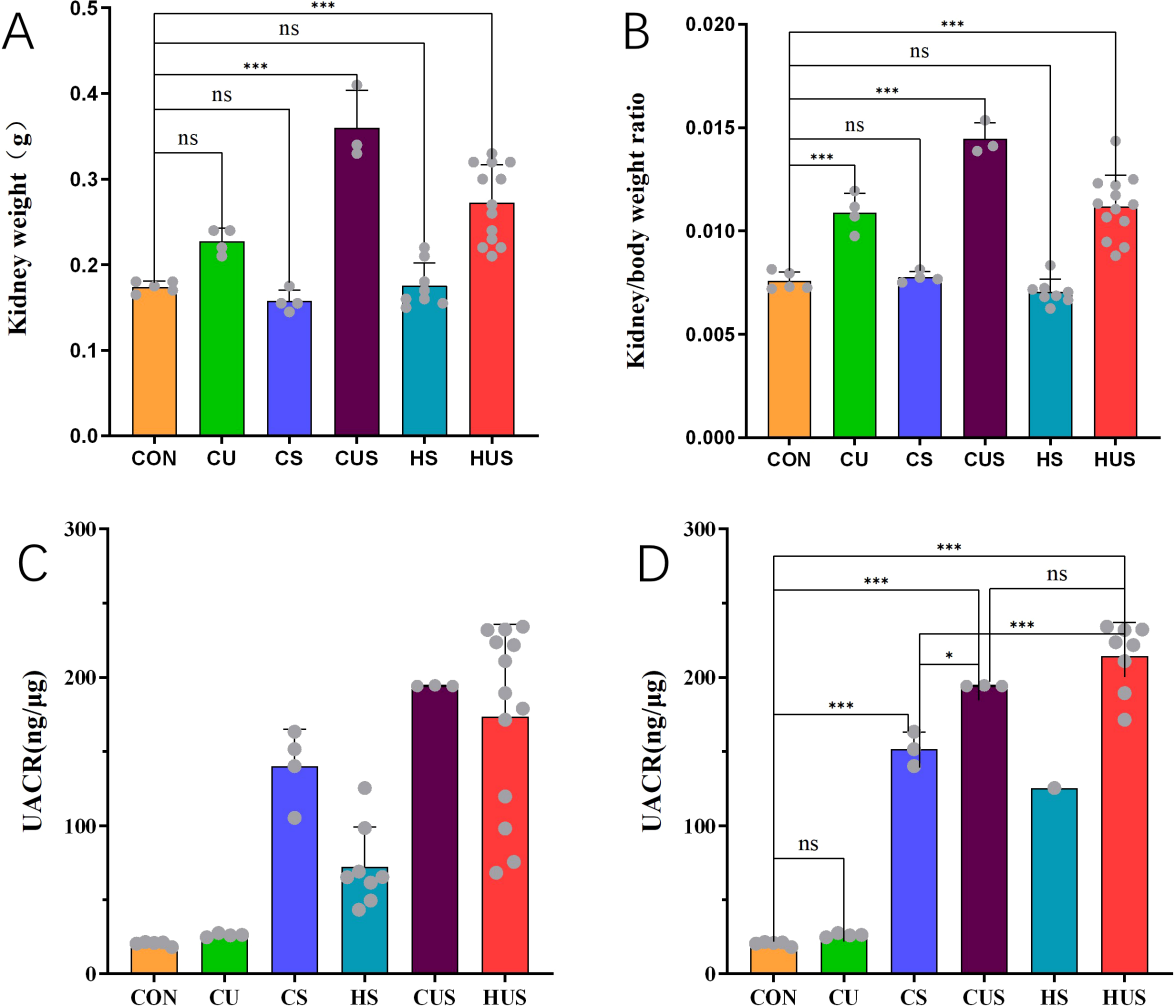
Kidney weight, kidney-to-body weight ratio, and UACR in each group of mice. **(A)** Comparison of kidney weight among the six groups (CON, CU, CS, CUS, HS, HUS). **(B)** Comparison of kidney-to-body weight ratio among the six groups. **(C)** UACR changes before sacrifice in each group. **(D)** UACR comparison among groups after excluding mice with recovered blood glucose. The HS group had only one remaining mouse due to glycemic recovery; therefore, this group was not included in the statistical analysis. **(A–C)** Sample sizes: CON (n=5), CU (n=4), CS (n=4), CUS (n=3), HS (n=8), HUS (n=13). **(D)** Sample sizes: CON (n=5), CU (n=4), CS (n=3), CUS (n=3), HS (n=1), HUS (n=8). Data are presented as mean ± SD. *P < 0.05, ***P < 0.001.

UN significantly increased kidney weight and the kidney-to-body weight ratio in CU, CUS, and HUS mice compared with CON (P < 0.001; **Figures 5A, B**). UACR was elevated in all STZ-treated groups (CS, CUS, HS, HUS) relative to both normal and UN controls (CON and CU) (**Figure 5C**). No significant difference in UACR was detected between CU and CON (P > 0.05). In contrast, CS, CUS, and HUS showed marked elevations compared with CON (P < 0.001), with UACR levels in CUS and HUS further exceeding those in CS (P < 0.05) (**Figure 5D**).

These findings suggest that UN alone caused compensatory hypertrophy of the remaining kidney without significantly altering UACR. By contrast, UN combined with diabetic induction accelerated DKD progression and further increased UACR.

#### 3.3.4 Renal histopathological changes in each group of mice

Light microscopy of CON mice showed intact glomerular architecture, no obvious hyperplasia was observed in the mesangial area, and the capillary loops were unobstructed. Ultrastructural analysis showed orderly podocyte foot processes, well-defined filtration slits, and a glomerular basement membrane of normal thickness.

In CU mice, the remaining kidney exhibited compensatory hypertrophy, with focal mesangial expansion, mild GBM thickening, and occasional localized podocyte foot process effacement. In CS and HS mice, glomerular capillary tufts were mildly enlarged, mesangial regions markedly expanded, mesangial cell proliferation evident, and some capillary lumens narrowed. Ultrastructural analysis revealed GBM thickening, podocyte foot process effacement, and reduced filtration slit density.

**Figure 6:**
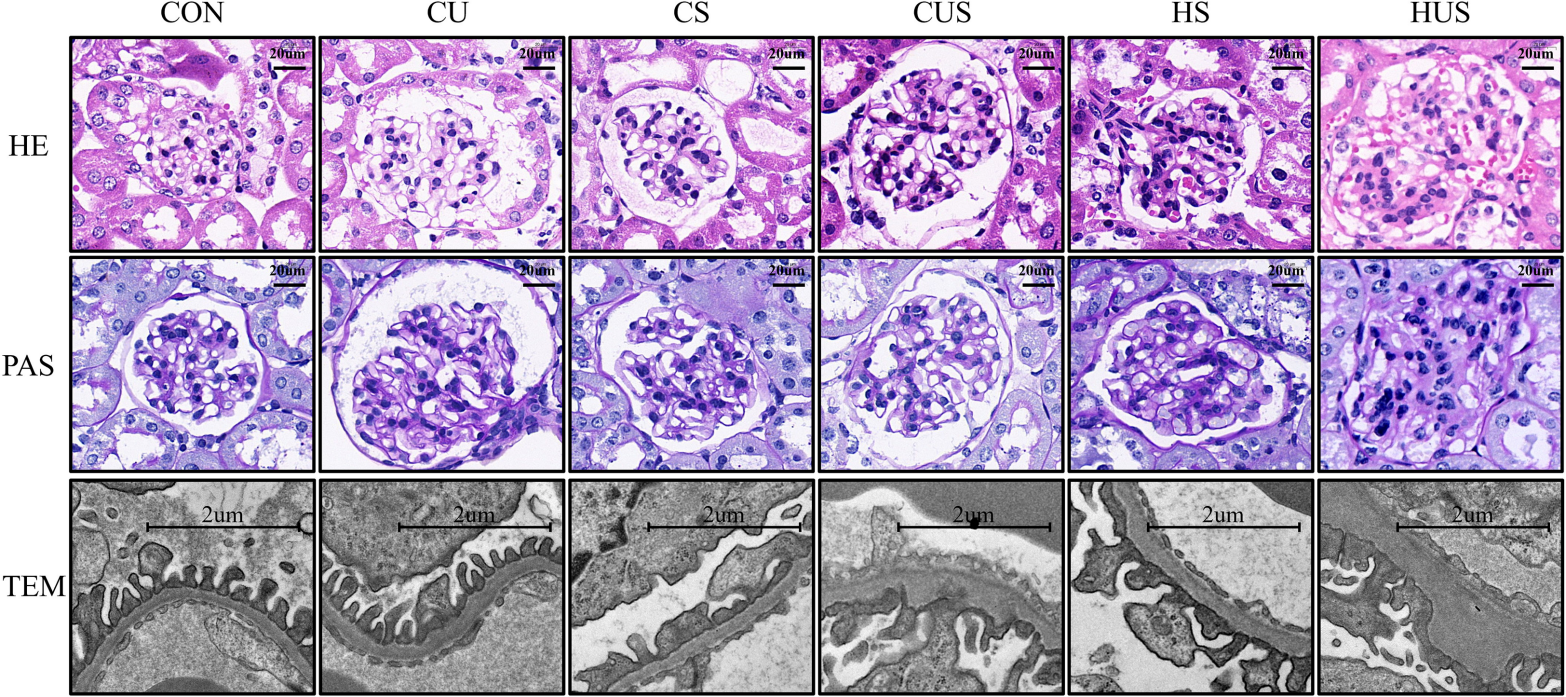
Histopathological analysis of mouse kidneys in each group. Kidney tissues were collected at 19 weeks post-modeling and examined by electron microscopy. Sections were stained with hematoxylin and eosin (HE) and periodic acid–Schiff (PAS). TEM: Transmission Electron Microscope.

CUS and HUS mice exhibited pronounced glomerular hypertrophy and extensive mesangial matrix expansion, accompanied by marked diffuse GBM thickening. HUS mice additionally showed focal glomerulosclerosis, widespread podocyte foot process effacement, and severe disruption of the glomerular filtration barrier, indicating the most severe glomerular injury among all groups. Pathological findings for each group are presented in **Figure 6**.

These findings indicate that UN induces compensatory hypertrophy in the remaining kidney; however, this hypertrophy differs from the early-to-mid-stage DKD-associated renal enlargement and glomerular tuft expansion, as it is not accompanied by diffuse mesangial proliferation. Furthermore, UN exacerbates renal pathology in DKD mice, and concurrent high-fat feeding further aggravates renal injury.

### 3.4 Comparative analysis of random blood glucose, body weight, kidney weight, kidney-to-body weight ratio, and UACR between diabetic mice and mice with glycemic recovery

During the establishment of the DM model, all STZ-injected mice initially reached the diabetes induction criterion (100%). However, as the disease progressed, a subset of mice failed to maintain sustained hyperglycemia and showed varying degrees of blood sugar recovery. To evaluate the impact of glycemic remission on disease phenotypes, mice were stratified according to their longitudinal blood glucose profiles. Specifically, 15 mice that maintained RBG levels ≥evel mmol/L throughout the study were classified as the diabetic mice with persistent hyperglycemia (DM), whereas 13 mice whose RBG levels declined below 16.7 mmol/L during the course were designated as the diabetic mice with spontaneous blood (NDM). Comparative analyses were then performed between the two groups.

**Figure 7:**
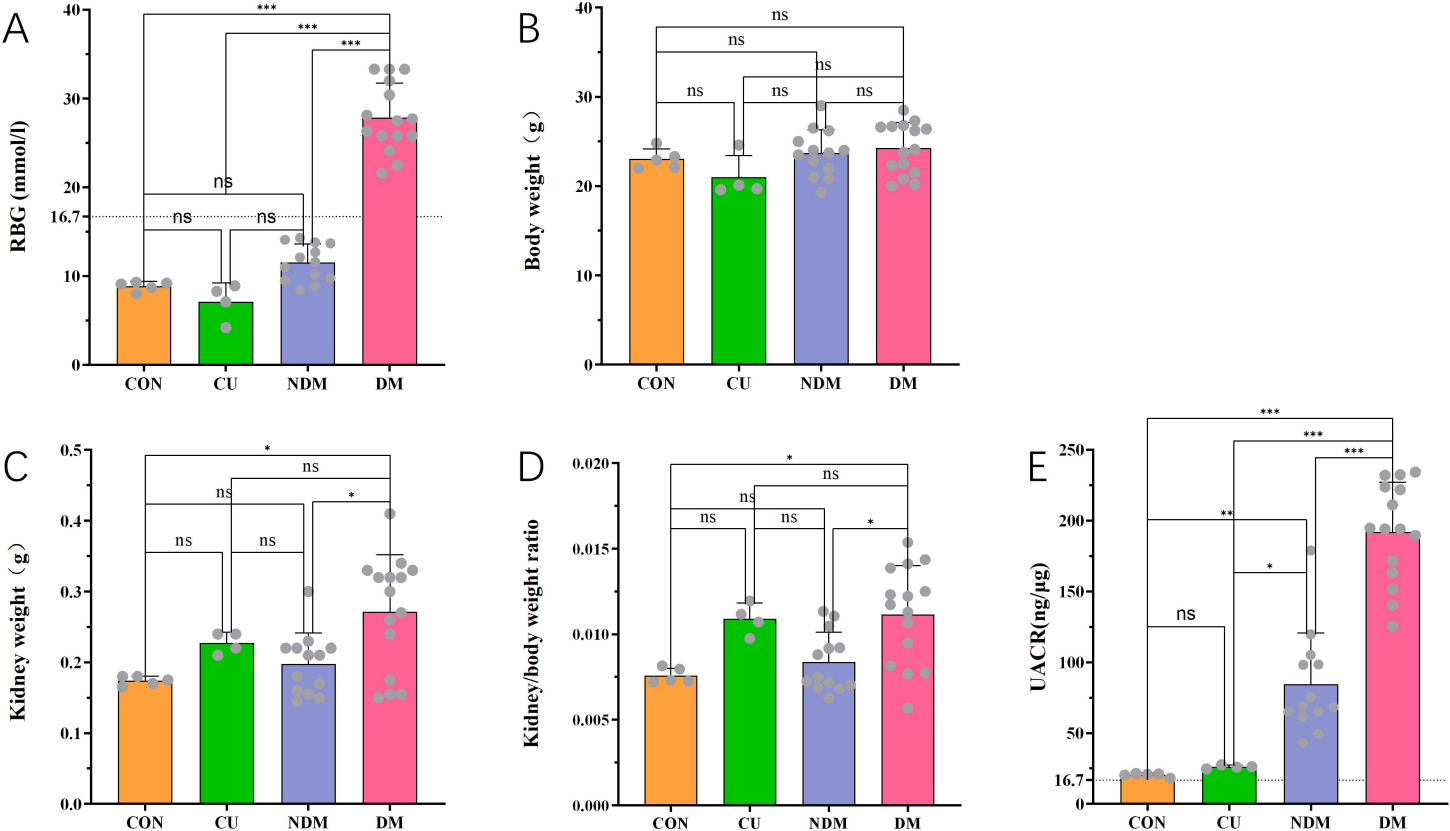
Comparison of random blood glucose (RBG), body weight, and UACR among DM, NDM, and control mice. CON, normal control; CU, unilateral nephrectomy control; DM, diabetic mice with persistent hyperglycemia; NDM, diabetic mice with spontaneous blood glucose recovery. **(A–E)** Sample sizes: CON (n=5), CU (n=4), NDM (n=13), DM (n=15). Data are presented as mean ± SD. *P < 0.05, **P < 0.01, ***P < 0.001.

Analysis revealed that RBG in the DM group was significantly higher than that in the CON, CU, and NDM groups (P < 0.001), whereas no significant differences were observed among the CON, CU, and NDM groups (P > 0.05) (**Figure 7A**). Body weight did not differ significantly among the four groups (CON, CU, NDM, DM; P > 0.05) (**Figure 7B**). Regarding renal parameters, the DM group exhibited markedly increased kidney weight and kidney-to-body weight ratio compared with the CON and NDM groups (P < 0.05), whereas no significant differences were observed among the NDM, CU, and CON groups (P > 0.05) (**Figures 7C, D**). UACR was significantly elevated in the DM group relative to the CON, CU, and NDM groups (P < 0.001), while the NDM group also showed a modest but significant increase compared with CON and CU (P < 0.05) (**Figure 7E**).

These results indicate that the spontaneous remission of blood glucose in some mice was accompanied by a reduction in renal injury, thus not showing typical characteristics of DKD, which was reflected in the decrease in UACR levels and the reduction of compensatory renal enlargement.

### 3.5 Analysis of factors affecting the establishment of induced diabetic mouse models

During the establishment of inducible DM mouse models, partial spontaneous remission of hyperglycemia was observed in some mice, compromising model stability. To investigate potential factors influencing blood glucose recovery, 31 mice receiving STZ injections were analyzed statistically (**Table 3**). The results demonstrated a significant association between modeling method and glycemic improvement in diabetic mice (Z = –2.209, P < 0.05). Specifically, mice receiving an initial STZ dose of 45 mg/kg/day combined with standard diet exhibited a markedly lower blood glucose recovery rate (14.3%) compared with those receiving 35 mg/kg/day STZ combined with a HFD (62.5%). In contrast, UN showed no significant correlation with blood glucose recovery (P > 0.05). These findings suggest that the modeling method is a critical determinant of DM mouse model stability. Although UN accelerates the progression of DKD, it does not improve the stability of hyperglycemia in DM mice.

**Table 3:** Analysis of factors associated with successful establishment of the diabetic mouse model.

Factor	Persistent hyperglycemia	Glycemic improvement	Z/F	P
Body weight at injection	23.80(22.30~26.20)	22.60(20.90~24.63)	-1.384	0.167
Pre-injection blood glucose	8.27 ± 0.80	7.68 ± 1.03	0.389	0.082
Modeling method (35mg/kg/d +HFD, %)	9 (60%)	15 (93.8%)	-2.209	0.027
Unilateral nephrectomy (Yes)	11 (73.3%)	7 (43.8%)	-1.641	0.101

The modeling method was classified as 35 mg/kg/day STZ combined with a HFD or 45 mg/kg/day STZ combined with standard diet.

## 4 Discussion

With the rising prevalence of diabetes worldwide, DKD has emerged as the most common microvascular complication and a leading cause of chronic kidney disease. Clinically, DKD is characterized by varying degrees of proteinuria and progressive renal dysfunction, with pathological features including mesangial expansion, diffuse podocyte foot process effacement, Kimmelstiel–Wilson nodules, glomerulosclerosis, as well as tubulointerstitial inflammation and fibrosis (17, 18). Elucidating its pathophysiological mechanisms and developing early interventions are critical to slowing disease progression, and the establishment of reliable animal models is a prerequisite for achieving this goal.

STZ–induced diabetes can be achieved by either a single high-dose or multiple low-dose injections. Previous studies have indicated that a single high dose of STZ exerts greater toxicity on the kidney, liver, and other major organs, often leading to severe hyperglycemic complications and increased mortality (19). In contrast, multiple low-dose STZ injections not only sustain hyperglycemia but also induce chronic insulitis and insulin deficiency, thereby mimicking autoimmune destruction of pancreatic β-cells (20–22). This strategy allows for a more reliable induction of insulin-dependent diabetes and its complications, including DKD, with renal injury more faithfully reflecting the consequences of chronic hyperglycemia. In B6 mice, intraperitoneal injections of STZ at 30–60 mg/kg/day for five consecutive days have been widely used to establish diabetes models (23–25).

HFD feeding, on the other hand, profoundly disrupts glucose and lipid metabolism, impairs hepatic and systemic metabolic homeostasis, and progressively induces hyperglycemia, hyperinsulinemia, and insulin resistanceinemia,NR that resemble dietary patterns and metabolic disturbances observed in type 2 diabetes mellitus (T2DM) patients (26–28). Long-term HFD feeding (approximately 20 weeks) alone has been reported to induce T2DM in B6 mice (29).

In this study, we employed STZ injection combined with HFD feeding and UN to evaluate model stability and factors contributing to DKD progression. In preliminary experiments, 45 mg/kg/day STZ reliably induced hyperglycemia in mice on a standard diet but resulted in severe hyperglycemic crises and markedly increased mortality in HFD+UN mice, suggesting that HFD may exacerbate STZ-induced glucotoxicity. To mitigate this risk, we reduced the STZ dose to 35 mg/kg/day in the formal experiment. Although this regimen induced initial hyperglycemia, it failed to sustain long-term hyperglycemia, with a notably higher recovery rate in HFD-fed mice (62.5%) compared with standard diet plus higher-dose STZ (14.3%). This observation suggests that insufficient STZ dosing may underlie model instability, potentially reflecting the robust βobust compensatory capacity of B6 mice (30). These findings underscore the critical role of dose optimization in balancing model stability against mortality risk.

Further analysis demonstrated that UN alone had no significant effect on baseline blood glucose or body weight, causing only compensatory hypertrophy of the remaining kidney without a significant increase in UACR or association with glycemic recovery. However, when combined with STZ-induced diabetes, UN significantly accelerated DKD progression, and the addition of HFD further exacerbated renal injury. These findings support the notion of synergistic interactions between metabolic stress and renal pathological damage.

It should be acknowledged that our study has certain limitations. First, while 45 mg/kg/day STZ caused excessive mortality in HFD+UN mice, 35 mg/kg/day failed to maintain stable hyperglycemia, resulting in a high rate of glycemic recovery (up to 88.9% in non-nephrectomized mice). Future work may therefore focus on fine-tuning STZ dosing within the 35–45 mg/kg/day range to better balance mortality risk with model stability. Second, only male C57BL/6J mice were included in our study. The absence of female mice restricts our ability to assess potential sex-specific differences in the onset and progression of diabetes and diabetic kidney disease. Future studies will incorporate both male and female animals to enhance the translational relevance and generalizability of the findings.

## 5 Conclusions

In summary, the injection dose of STZ may be the main factor affecting the stability of the DM model in C57BL/6J mice, and subsequently influencing the degree of kidney injury. Insufficient dosage can easily lead to spontaneous remission of blood glucose and weaken the stability of the model. Although UN does not impact DM model stability, it markedly accelerates DKD progression and exhibits a synergistic effect with HFD. Thus, an appropriate dose of STZ combined with UN and HFD is an optimal strategy for constructing an STZ-induced DKD model in C57BL/6J mice, effectively recapitulating the clinical and pathological features of human DKD and providing a reliable platform for mechanistic studies and therapeutic intervention.

## Data Availability

The raw data supporting the conclusions of this article will be made available by the authors, without undue reservation.
